# State of the Art in Temporomandibular Joint Arthrocentesis—A Systematic Review

**DOI:** 10.3390/jcm12134439

**Published:** 2023-06-30

**Authors:** Marta Siewert-Gutowska, Rafał Pokrowiecki, Artur Kamiński, Paweł Zawadzki, Zygmunt Stopa

**Affiliations:** 1 Department of Cranio-Maxillofacial Surgery, Oral Surgery and Implantology, Medical University of Warsaw, 02-005 Warsaw, Poland; martasiew@gmail.com (M.S.-G.);; 2 Private Practice, Prive Esthetic and Facial Feminization Surgery, 02-640 Warsaw, Poland; 3 Department of Transplantology and Central Tissue Bank, Medical University of Warsaw, 02-004 Warsaw, Poland

**Keywords:** temporomandibular, arthrocentesis, maxillofacial, TMJ, pain, hyaluronic acid

## Abstract

Temporomandibular joint disorders are a heterogenic group of clinical conditions, which impair physiological functioning of the masticatory system. Arthrocentesis of the temporomandibular joint has become a widely approved method for non-invasive treatment, bridging the gap between conservative and surgical approaches. Regardless of technique, treatment is based upon joint lavage and lysis of the inflammatory fibrous tissue adhesions, which, in turn, improves joint mobility and reduces pain and closed lock. Recently, approaches for intra-articular injections have been proposed as adjuvant or replacement therapy. The aim of this study was to assess the most efficient technique of arthrocentesis. A systematic search based on PRISMA guidelines, including a computer search with specific keywords, a reference list search and a manual search, was performed. Relevant articles were selected after three search rounds for final review. The studies pulled for the analysis presented information about the relevant predictors, including the technique of arthrocentesis (single- or two-needle method), fluid used for lavage (Ringer lactate or saline), volume of the fluid, application of the injectable, number of interventions, pain (VAS) and mouth opening scores (MMO) and follow-up. All cohorts showed improvement in mouth opening, but significant pain reduction was observed only in cohorts treated either by arthrocentesis alone or arthrocentesis followed by intra-articular injectables. Intra-articular injectables used alone failed to reduce pain post-operatively when compared to other cohorts. We concluded that both double-needle and single-puncture arthrocentesis techniques are equally efficient. Application of the adjuvant injectable did not improve the outcomes of arthrocentesis performed alone. The volume of the fluid used for joint lavage and its chemical composition were not significant in clinical outcomes. However, due to the lack of homogeneity in the study settings, a meta-analysis could not be applied and a systematic review was conducted. We still, however, state that there is a knowledge gap in the current literature regarding the use of injectables alone, as well as a longitudinal follow-up, which provides information about treatment efficiency. More high-quality and randomized controlled trials are required to shed light on this subject.

## 1. Introduction

Arthrocentesis of the temporo-mandibular joints (TMJ) has been described as a proven treatment modality, filling the gap between conservative and operational approaches to temporo-mandibular disorders (TMD) [1,2,3]. Among the most recently reported TMDs as a rationale for intra-articular lavage are as follows: internal derangements, e.g., disc displacement with or without reduction (DDWOR), osteoarthritis being a cause of masticatory pain (at rest and/or during chewing) and limitation in maximum mouth opening, which significantly reduces quality of life, impairs oral intake, results in the failure of the masticatory system and problems with teeth and gums [4].

Until now, several techniques of arthrocentesis (AC) have been described, of which double-needle lavage and single-puncture techniques are considered the gold standard of TMJ AC. Originally, the two-needle technique was described by Nitzan et al. in 1991 [5], while the single-puncture method was a simplifying modification of the former, designed and developed by Singh and Varghese in the early 2010s [6].

The idea of the double-needle technique of intra-articular lavage is to remove inflamed synovial fluid, which is why a volume of 100–500 mL of saline/ringer is usually used. However, the basis for the single-needle technique is more to induce intra-articular pressure, to perform a hydrodissection of the inflamed tissue and remove the adhesions that block/interrupt the normal functioning of the TMJ. For the past few decades, these two techniques have been developed, modified and crossed over. Therefore, diversified results have been published as different fluids (saline/ringer) with a varying volume of the total solution used for lavage and were applied at different intervals (from a single procedure to several, until final follow-up) [3,7,8,9]. In addition, many protocols added an intra-articular drug deposition (IDD) as an adjuvant therapy or even a substitute to AC, thus making intracapsular injections a standalone procedure. Different IDD proposals have been described, of which hyaluronic sodium, platelet-rich plasma/fibrin or dexamethasone are the most frequently proposed. However, there are studies implying that the intra-articular placement of our own blood or fat-derived stem cells are promising, thus making the protocols for TMDs extremely diversified [10,11,12,13].

Therefore, scientific and evidence-based protocols are required to fully appreciate the variables available and to choose the best treatment option for specific patients. Within this systematic review, the aim is to estimate whether the double- or single-puncture technique provides a better clinical outcome, to elaborate if fluid type and its volume present clinical significance, and if the application of IDD is needed to achieve better results.

## 2. Materials and Methods

In order to meticulously evaluate TMJ arthrocentesis in clinical conditions, the following study design was proposed, according to PICO guidelines (Table 1).

**Figure 1:**
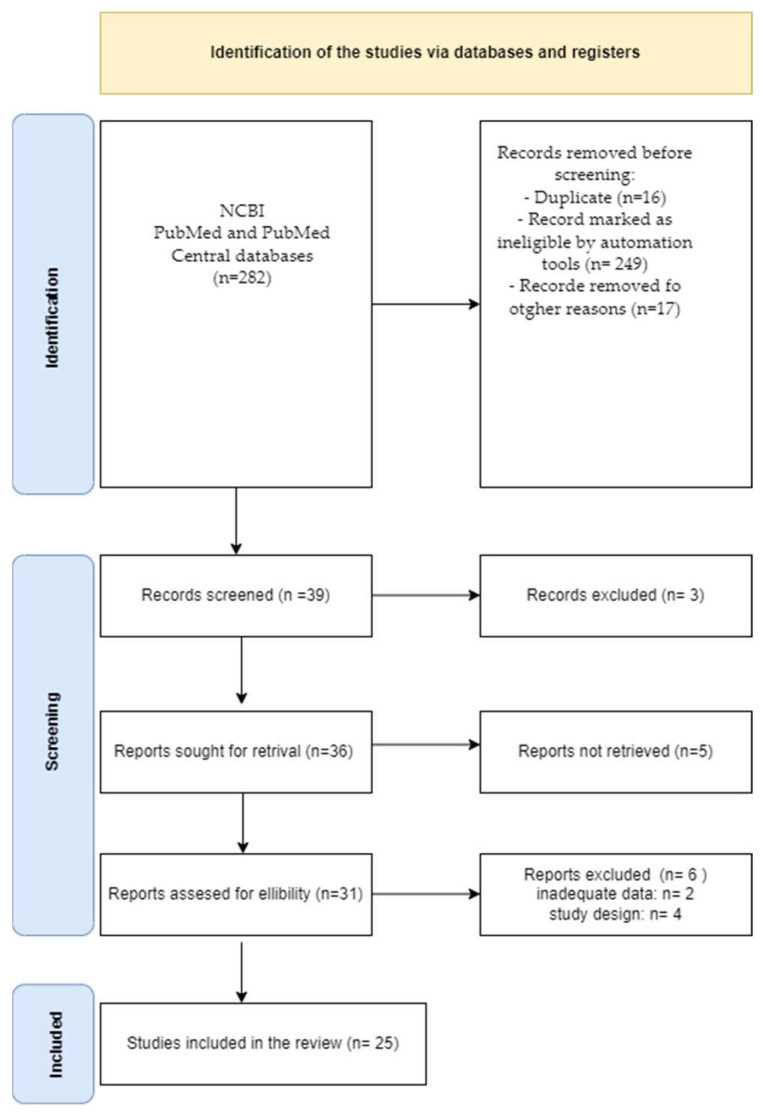
Search strategy of the original studies meeting the criteria of the review, according to PRISMA guidelines. Among 282 articles, 25 original clinical studies met the criteria for the analysis.

A ssystematic review of the literature was performed, according to PRISMA guidelines. After formulating the research questions and objectives, a NCBI PubMed and PubMed Central databases search was conducted, between January 2012 and April 2022. The existing literature, based on TMJ arthrocentesis techniques in humans, was searched, screened for inclusion, assessed with regard to quality, extracted and analyzed as described in the flowchart (Figure 1) [14]. The phrases “TMJ” OR “temporomandibular” AND “arthrocentesis” OR “lavage” OR “lysis” were used. The inclusion criteria in the study were taken from the original study and were as follows: the available full text, information on the performed arthrocentesis, information about the cohort number, the evaluation of pain reduction in the VAS scale, the evaluation of maximal mouth opening measured in millimeters, follow-up after at least 1 month, the number of total interventions, information about the fluid used for the arthrocentesis and the volume used and information about intracapsular drug injection. The exclusion criteria were as follows: review or narrative studies, experimental, in vitro or animal model studies, original studies where any surgery was performed earlier or as control groups and inadequate data not meeting the inclusion criteria. Studies published only in English in the last 10 years were included in the study.

Statistical analysis was performed with IBM SPSS Statistics v. 25 using an analysis of variance. A *p*-value of <0.05 was considered statistically significant in all of the tests. Both paired and unpaired *t*-tests were used to compare pre-treatment and post-treatment differences in MMO, level of pain and level of dysfunction. This study was not registered and therefore there is no registration number.

## 3. Results

Among the 265 articles reviewed, 25 original publications evaluating arthrocentesis in temporo-mandibular disorders were selected and included in the analysis (Figure 1) (Table 2 and Supplementary Table S1).

The research included in the analysis and retrospective studies, observational or case–control studies varied between cohorts, method of arthrocentesis, fluid used for lavage and/or intracapsular lysis of the attachments or intra-articular drug deposition thereafter (Supplementary Table S1). The mean age of the patients, who, in the collected studies, had disc displacement with/or without reduction (DDWOR), was 39.5 years old, of which females were more frequently reported. The two most relevant clinical indicators of disease severity at the study’s starting point and result at the final follow-up after treatment were analyzed and included pain (the VAS scale) and maximum mouth opening (MMO). The type of treatment, fluid used, technique of application and the use of intracapsular drug deposition were investigated, in order to estimate the most efficient method of achieving a DDWOR-associated reduction in symptoms. The outcomes in a total of 548 interventions with the use of arthrocentesis and 480 interventions of arthrocentesis followed by intra-articular deposition of the drug were compared. In total, 71 interventions of intra-articular injection of a medicine as an alternative to AC were evaluated (Table 3). The double-needle technique, according to [5], was the most frequently favored technique over the single-needle technique in the analyzed material (Supplementary Table S1).

**Figure 2:**
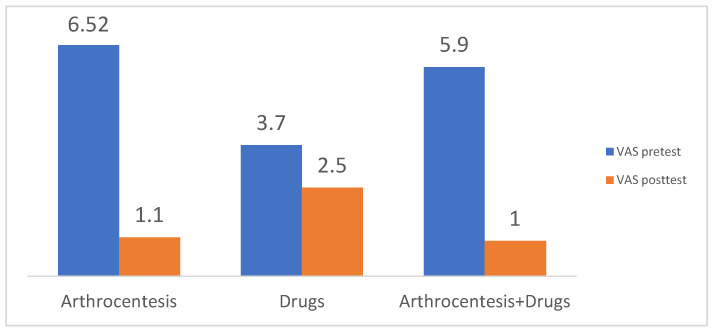
Chart showing the significant reduction in VAS in cohorts treated either with arthrocentesis or arthrocentesis supplemented with intra-articular injection. No significant reduction in VAS was reported in the cohorts treated only with intracapsular deposition of the medication (*p* < 0.05).

**Figure 3:**
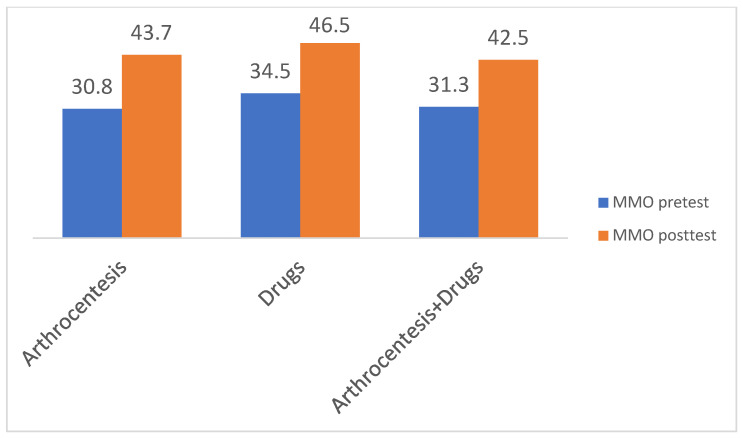
Chart showing a significant increase in cohorts treated either with arthrocentesis or arthrocentesis supplemented with intra-articular injection. Contrary to VAS changes, in the group treated with injectables, significant improvement in mouth opening was observed. Differences in MMO change after intervention were not statistically significant in paired *t*-tests (*p* > 0.05).

Arthrocentesis (double and single technique) was found to be statistically significant in the reduction in pain (VAS) from the mean pre-operative value of 6.52 to 1.60 at the final follow-up (*p* = 0.00) (Supplementary Table S1). This provided a decrease in reported pain in the VAS scale of 4.92 (Figure 2). In addition, it yielded significantly better mouth opening from a mean of 30.8 mm to 43.73 mm (*p* = 0.00) (Figure 3). Arthrocentesis, with the subsequent application of an intra-articular injection of a drug (PRP, HA or other), was found to be another efficient method of treatment, which provided a VAS reduction from a mean of 5.9 to 1 (*p* = 0.00) (a VAS decrease of 4.9) and an increase in mean MMO from 31.3 mm to 42.5 mm (*p* = 0.00). VAS reduction was comparable between the AC and AC + drug groups, without statistically significant differences in the independent sample *t*-tests.

**Figure 4:**
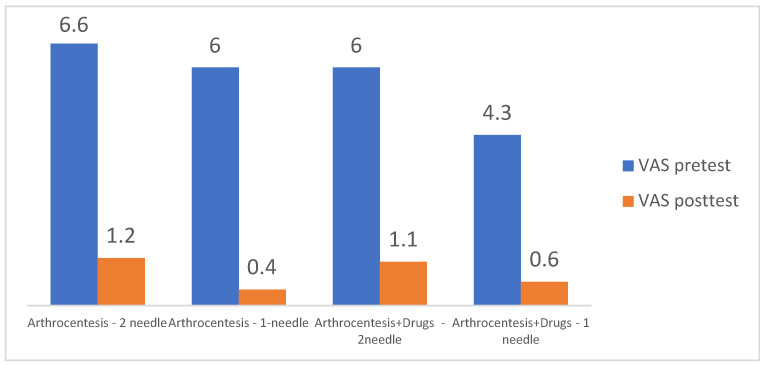
Chart showing VAS reduction according to the type of arthrocentesis (double-needle VS single-needle technique) with or without the application of injectables. All variants of the treatment provided a significant reduction in pain at the endpoint of the studies (*p* = 0.001) without significant differences in paired *t*-tests between treatment protocols (*p* > 0.05).

Resolution of the pain and better mouth opening were significant in both the double-needle and single-needle techniques, without significant differences between these two techniques in the independent sample *t*-tests. (Figure 4).

**Figure 5:**
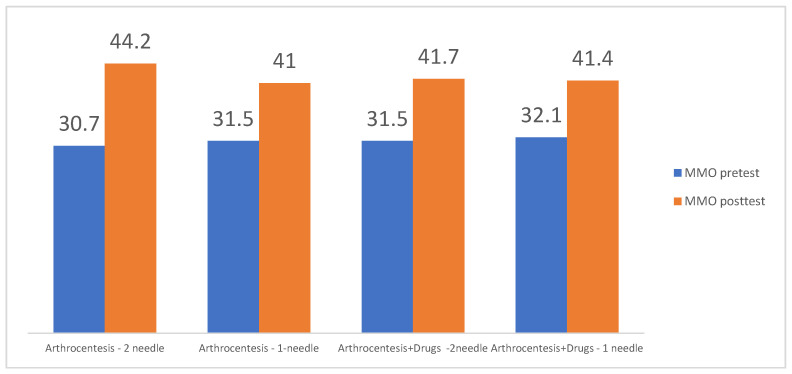
Chart showing improvement of maximum mouth opening (MMO) according to type of arthrocentesis (single-needle VS double-needle technique) with or without the application of injectables. All variants of the treatment provided significant improvement at the endpoint of the studies (*p* = 0.001) without significant differences in paired *t*-tests between treatment protocols (*p* > 0.05).

The most frequently used drug/medications for intra-articular injections were hyaluronate acid, steroids, PRP/PRF or combinations of mean volume; 1–2 mL deposed into the upper level of the joint, sometimes with pericapsular deposition (Supplementary Materials). However, due to the diversity of the methodology, statistical analysis of which drug is the most efficient was not possible. In 24 studies, a single intervention of TMJ was performed until the final follow-up (usually at sixth months). In five studies, patients underwent more than one intervention of joint lavage/lysis (3–5) of AC until follow-up. Clinical improvement was, therefore, not related to the number of interventions. Single treatment (arthrocentesis with or without drug injection) was found to be effective in most studies, significantly reducing pain and increasing mouth opening at four weeks post-op. Both arthrocentesis and arthrocentesis supplemented with drug injection were found to be efficient. No statistically significant differences were found between VAS and MMO improvement between these groups (Figure 5). Likewise, the fluids used for intra-articular lavage (0.9% NaCL vs. Ringer lactate) were not significant at the endpoint of the studies and so not included in the analysis. The total volume of the fluid used for joint lavage was found to be statistically insignificant.

In two studies (a total of 71 interventions), intra-articular drug injection without AC was used as a control cohort. The reported reduction in VAS and increase in MMO similar to AC alone showed a promising alternative for articular lavage; however, due to the small control cohort, these results could not be statistically compared in a paired *t*-test with groups treated either with the AC or AC + drug. However, due to the lack of homogeneity in the study settings, a meta-analysis could not be applied and a systematic review was carried out.

## 4. Discussion

Temporomandibular disorders (TMDs) are a group of complex and diversified clinical conditions, of which the most frequently reported symptoms are pain in the temporo-auricular region and limited mouth opening (closed lock).

Disc displacement treatment varies from conservative methods to surgical, of which the latter is the final approach. Orthodontic appliances or prosthetic splints are still the first line of treatment regarding functional temporomandibular disorders (TMD) [35]. Only advanced and non-responding to conservative treatment cases of severe TMJ malfunction syndromes were taken into consideration for advanced surgery, starting from classic eminectomy, the LeClerk procedure, disk anchoring and, finally, total joint replacement, with the use of stock or a custom-made TMJ prosthesis [36,37,38]. As a result, an approach towards a less invasive strategy, thus being a bridge between conservative and surgical techniques, was initially introduced by [5] and has been developed by other authors ever since. In its original formula, TMJ arthrocentesis was adapted from other specialties and was based on the idea of an injection of two needles into the upper level of the joint and its lavage with the use of 200 mL of Ringer lactate within 15–20 min, with the use of an infusion bag placed 1 m above the patient’s joint. The idea was to remove the inflammatory intracapsular fluid and carry out lysis of the adhesions through hydraulic pressure of the upper floor of the TMJ, giving results similar to arthroscopy. The cohort evaluated by [5] was diagnosed with acute closed lock, non-responsive to a conservative treatment and primarily qualifying for surgery. Their study, however, as a first, proved that arthrocentesis may be a beneficial and efficient way to improve mouth opening and to reduce pain, with a low level of post-operative complications, without the need for direct-vision TMJ lysis and disc reposition, thus bridging the gap between non-surgical and surgical treatment [5,39,40].

With time, joint lavage under local anesthesia was introduced into clinical practice and indications for such were expanded. Nowadays, young patients with pain and clicking are taken into consideration, as well as elderly people with diagnosed osteoarthritis as groups of individuals that would benefit from TMJ arthrocentesis. In 2013, Singh and Vargese [6] introduced a novel adaptation of the classic method, where two 18-gauge needles were connected using orthodontic solder, thus providing two-way fluid flow through a one-puncture entry point to the capsule. Then, 200 mL of normal saline was used for TMJ lavage, which decreased pain and improved mouth opening. Their method, frequently described as a single-puncture TMJ lysis, was also used by other authors with good results, compared to Nitzan’s technique.

It appeared that the choices of methods presented in the analyzed studies were operator/department-preferred assumptions between either single-puncture or double-needle procedures. Only one study compared these techniques within the cohort [26]. The authors showed that single-puncture techniques provide better outcomes than double-needle procedures, where the difference in the width of mouth opening between the concentric needle technique and the two-needle technique, at 3- and 6-month intervals, was found to be statistically significant (44.91 mm for single-needle and 37.36 mm for the double-needle technique, respectively).

Following the tatistical analysis of the studies that met the inclusion criteria and with the total number of interventions taken into consideration, the double-needle technique provided a MMO of 44.2 mm, while a single puncture, 41 mm of maximal mouth opening, did not show a statistically significant difference between these two methods within 6 months following the operation. Thus, it can be concluded that both methods of TMJ lavage/lysis are efficient and may be used to the operator’s preference. In addition, pain reduction was comparable between these two techniques, as the double technique showed a VAS reduction from a mean value of 6.6 to 1.2 and the single-puncture technique showed a reduction from a mean value of 6.0 to 0.4, also without significant differences in the paired *t*-tests. Thus, concluding that both the single-or double technique are similarly efficient in the reduction in pain and increasing mouth opening in patients with DDWOR [3,6,7,8,9,16,17,18,19,21,22,23,24,25,26,28,29,30,31,32,33,34]. Increased values of MMO following the operation interfered with the more decreased VAS reported by patients in the analyzed material. These observations are consistent with the observations of other authors [18]. Additionally, the manual lysis of adhesions with the use of a needle during arthrocentesis may be beneficial in increasing post-operative MMO, according to [21].

Intracapsular drug deposition is another approach used as an addition to TMJ arthrocentesis or used as a stand-alone treatment. Various studies have stated that an additional injection of either HA, DXM, HA + DXM, PRP or IPRF is superior to arthrocentesis or may be used as a beneficial addition to conservative treatment, which is frequently not effective [4,17,19,29].

In the analyzed material, 480 interventions of arthrocentesis with the use of a drug were evaluated in comparison to 548 interventions of arthrocentesis (both single- and double-needle procedures). Application of the drug after arthrocentesis did not significantly improve the outcomes during follow-up. Both groups represented satisfying clinical improvement in the tested cohorts.

Due to a diversified methodology and the medications being used, it was not possible to statistically evaluate which drug used is the most efficient when used intracapsularly. It is generally stated that HA is superior to steroids, as it does not induce potential bone resorption, as was observed in cases treated with the latter. In addition, PRF and IPRF were described as more efficient in pain reduction than steroids. In the study [41], methyloprednizolone was not found to be superior to saline in pain reduction [41]. Overall, the AC+ drug group appeared insignificantly superior to arthrocentesis alone. Similar observations were described in another systematic review, but due to the heterogenicity of the studies, a meta-analysis of which injectable is the most efficient was not possible [42].

In the analyzed material, only two studies (71 interventions) evaluated intracapsular drug injection as the sole method for the treatment of DDWOR. Despite the fact that in the overall analysis of the cohort, it was found that the mean VAS may have decreased from 3.7 to 2.5 and MMO increased from 34.5 mm to 46.5 mm, this suggested that these studies represent significant commentaries. In the study of [17], pain reported by patients increased post-operatively from a mean of 1.7 to 4.07, failing to decrease pain when chewing. According to the authors, arthrocentesis treatment prior to PRP injection can achieve a more satisfactory outcome than the use of a drug alone. On the contrary, in a study by [19], VAS was significantly reduced from 5.1 to less than 1 (also with the use of PRP). However, in both studies, significant MMO was achieved after drug injection [17,19]. A small cohort and diversified results were insufficient to perform statistical analysis comparing the drug-only approach with AC and AC+ drug cohorts.

A follow-up period is crucial for the assessment of therapy success and its maintenance. However, among the existing literature, a relatively short observational period is presented. Only one study presented a longitudinal follow-up of 96 months [22]. In three studies, follow-up was performed up to 12 months after the procedure [23,29,33]. Other studies presented early outcomes (from one up to 6 months) with a single intervention providing information that arthrocentesis performed either by the double-needle lavage technique or single-puncture lysis was able to significantly reduce reported TMJ pain and increase mouth opening at follow-up. In only one systematic review, it was suggested that the execution of multiple sessions (three to five) is superior to a single session. Such observations were not confirmed within this study. As there were no statistically significant differences between the obtained results and the follow-up, due to heterogenous study designs, caution is required when interpreting the meta-analyses. It seems that more longitudinal observations should be performed when assessing the efficiency of arthrocentesis, as well as the number of interventions needed to reach satisfying clinical improvement [43].

The total volume of the fluid used for arthrocentesis ranged from 2 mL up to 500 mL between different studies. However, the volume of the fluid used was not statistically significant in the level of clinical improvement in the paired and unpaired *t*-tests. There has been a debate on whether Ringer lactate or saline is more effective in joint lavage/lysis. This is due to the biochemical composition and pH of the fluids. Ringer lactate is the most comparable to human serum. Among the analyzed studies, Ringer lactate and 0.9% saline were used. However, the statistical analysis showed no differences between these two irrigants in the efficiency of TMJ arthrocentesis.

## 5. Conclusions

Based on the pre-existing literature, arthrocentesis, performed either through the double-needle technique or the single-puncture method, is a clinically proven form of treatment and is significant in decreasing pain and increasing the maximum width of mouth opening in disc displacement with or without reduction. Additional intra-articular application of medication, such as hyaluronic acid, dexamethasone, PRP/PRP and others, does not improve the outcome of arthrocentesis. However, using intra-articular injections with medications without arthrocentesis is less effective. It provides comparable MMO, but is less effective at reducing pain. The type of fluid used for lavage and lysis (ringer lactate or saline), as well as the total volume of the fluid used, are not significant. However, more studies with longer follow-ups are necessary to estimate which approach provides the most efficient and stable results in DDWOR treatment.

## Figures and Tables

**Table 1:** Proposed study design with reference to participants, interventions, comparisons and outcomes.

Description	Main Objective
**Patient**	Disc displacement with or without reduction
**Population**	Generally healthy. Treated previously by means of a conservative approach or by no one
**Problem**	Pain at rest/chewing presented in the VAS scale pre- and post-op, limited mouth opening presented in milimeters pre- and post-op
**Intervention**	Arthrocentesis of the temporomandibular joint (single-needle, double-needle procedure or both)
**Comparison**	Arthrocentesis with intracapsular iniectables or injection only
**Outcome**	% of bone resorption with regard to either SP or SA or SH

**Table 2:** Studies included in the analysis. RCT-randomized clinical study. Quality of the study based on Swedish council’s guidelines on technology assessment in healthcare (SBU), where Grade A: randomized clinical study or a prospective study with a well-defined control group, defined diagnosis and end points, diagnostic reliability tests and reproducibility tests described, blinded outcome assessment; Grade B: moderate value of evidence. The following criteria should be met: cohort study or retrospective case series with defined control or reference group, defined diagnosis and end points, diagnostic reliability tests, and reproducibility tests described. Grade C: low value of evidence. One or more of the following conditions: large attrition, unclear diagnosis, and end points and poorly defined patient material [15].

Authors	Study Design	Cohort Evaluated	Mean Age	Quality of the Study
Grossman (2019) [16]	Retrospective	234	33.02	B
Lin et al. (2018) [17]	Retrospective	90	40.7	B
Rossini et al. (2021) [18]	Retrospective	72	32.46	B
Chandra et al. (2021) [19]	Clinical	52	30.65	B
Grossman et al. (2017) [20]	RCT	26	40.55	A
Lee et al. (2021) [21]	Prospective	40	46.6	
Yapıcı-Yavuz et al. 2018 [22]	Prospective	44	n/a	B
Kim et al. (2019) [23]	Retrospective	57	38.65	A
Ghoneim et al. (2021) [24]	Prospective	40	27	B
Altaweel et al. (2020) [25]	RCT	32	28.02	B
Kumar et al. (2020) [26]	Prospective	40	25.18	A
Santagata et al. (2020) [27]	Clinical	28	40.6	A
Rao et al. (2019) [28]	Prospective	20	37	B
Giraddi et al. (2012) [29]	Prospective	8	26.88	B
Giraddi et al. (2012) [29]	Prospective	8	26.88	B
Andrabi et al. (2019) [30]	Prospective	50	28.96	B
Sequeira et al. (2019) [31]	Prospective	10	38.40	B
Gorrela et al. (2017) [8]	RCT	62	42.3	B
Chandrashekhar et al. (2015) [7]	Prospective	50	41.5	B
Giraddi et al. (2015) [32]	Prospective	14	30.4	A
de Riu et al. (2013) [33]	Prospective	30	43.5	B
Sharma et al. (2013) [3]	RCT	20	25	B
Singh & Vargese (2013) [6]	Prospective	20	44	A
Thomas et al. (2012) [9]	Prospective	32	23	B
Gurung et al. (2017) [34]	Prospective	20	39	B

**Table 3:** Mean values and standard deviations of VAS and MMO changes in the three approaches to DDWOR treatment taken from 25 publications included in the study, which cover 548 interventions of arthrocentesis, 480 interventions of arthrocentesis followed by intracapsular injectables and 71 interventions of injectables only. Evidence level based on the work of [15], where Level 1—strong (at least two studies assessed with level ‘A’), Level 2—moderate (one study with level ‘A’ and at least two studies with level ‘B’), Level 3—limited (at least two studies with level ‘B’), Level 4—inconclusive (fewer than two studies with level ‘B’) [15].

	Total Number of Interventions	VAS Pre	VAS Post	MMO Pre (mm)	MMO Post (mm)	Evidence Level
**Arthrocentesis**	548	6.52 ± 1.60	1.10 ± 0.98	30.8 ± 4.85	43.70 ± 4.19	2
**Arthrocentesis + drug**	480	5.90 ± 1.97	1 ± 0.64	31.3 ± 5.79	42.50 ± 3.18	1
**Drug only**	71	3.7 ± 2.82	2.5 ± 2.17	35.95 ± 0.21	44.8 ± 1.34	3

## Data Availability

Not applicable.

## Supplementary Materials

The following supporting information can be downloaded at: https://www.mdpi.com/article/10.3390/jcm12134439/s1.
